# Supplementary material for: Virtual reality therapy in managing cancer pain in middle-aged and elderly: a systematic review and meta-analysis
Source: PeerJ. 2024 Dec 13;12:e18701. doi: 10.7717/peerj.18701 (PMC11648695; doi:10.7717/peerj.18701)
Supplement: Supplemental Information 3 [file peerj-12-18701-s003.docx]

**Supplementary Table 1 Bias-dependent risk assessment**

| Author/Year | Random sequence generation | Allocation concealment | Blinding of participants and personnel | Blinding of outcome assessment | Incomplete outcome data | Selective reporting | Other bias |
| --- | --- | --- | --- | --- | --- | --- | --- |
| Gao  (2022) | Computer random | Unclear | N | Y | Complete | N | Unclear |
| Turrado  (2021) | Computer random | Airtight envelope | N | Y | Complete | N | Unclear |
| Feyzioglu  (2020) | Computer random | Airtight envelope | Y | Y | Complete | N | Unclear |
| Basha  (2022) | Computer random | Airtight envelope | Unclear | Y | Complete | N | Unclear |
| Mohammad  (2018) | Computer random | Unclear | N | Y | Complete | N | Unclear |
| Villumsen  (2019) | Random number table | Airtight envelope | Y | Y | Complete | N | Unclear |
| Zhang  (2022) | Computer random | Electronic document | N | Y | Complete | N | Unclear |
