# Supplementary material for: Virtual reality therapy in managing cancer pain in middle-aged and elderly: a systematic review and meta-analysis
Source: PeerJ. 2024 Dec 13;12:e18701. doi: 10.7717/peerj.18701 (PMC11648695; doi:10.7717/peerj.18701)
Supplement: Supplemental Information 4 [file peerj-12-18701-s004.docx]

**Supplementary Table 2 Scores assessing the methodological quality**

| Eligibility criteria  specified | Gao  (2022) | Turrado  (2021) | Feyzioglu  (2020) | Basha  (2022) | Mohammad  (2018) | Villumsen  (2019) | Zhang  (2022) |  |  |
| --- | --- | --- | --- | --- | --- | --- | --- | --- | --- |
| Random allocation | 1 | 1 | 1 | 1 | 1 | 1 | 1 |  |  |
| Concealed  allocation | 0 | 1 | 1 | 1 | 0 | 1 | 0 |  |  |
| Groups similar at  baseline | 1 | 1 | 1 | 1 | 1 | 1 | 1 |  |  |
| Subject blinding | 0 | 0 | 1 | 0 | 0 | 0 | 0 |  |  |
| Therapist blinding | 0 | 0 | 0 | 0 | 0 | 1 | 0 |  |  |
| Assessor blinding | 1 | 1 | 1 | 1 | 1 | 1 | 1 |  |  |
| Less than %15  dropouts | 1 | 1 | 1 | 1 | 1 | 1 | 1 |  |  |
| Intention to treat  analysis | 1 | 1 | 1 | 1 | 1 | 1 | 1 |  |  |
| Between-group  statistical  comparisons | 1 | 1 | 1 | 1 | 1 | 1 | 1 |  |  |
| Point measures and  variability data | 1 | 1 | 1 | 1 | 1 | 1 | 1 |  |  |
| Total PEDro score | 7/10 | 8/10 | 9/10 | 8/10 | 7/10 | 9/10 | 7/10 |  |  |
|  |  |  |  |  |  |  |  |  |  |
