# Supplementary material for: Virtual reality therapy in managing cancer pain in middle-aged and elderly: a systematic review and meta-analysis
Source: PeerJ. 2024 Dec 13;12:e18701. doi: 10.7717/peerj.18701 (PMC11648695; doi:10.7717/peerj.18701)

Study or Subgroup	Experimental Mean	Experimental SD	Experimental Total	Control Mean	Control SD	Control Total	Weight	Mean Difference IV, Random, 95% CI
Basha	41.23	10.3	30	51.17	10.45	30	13.4%	-9.94 [-15.19, -4.69]
Feyzioglu	1.53	1.3	19	2.16	1.72	17	0.0%	-0.63 [-1.64, 0.38]
Mohammad	0.33	0.72	38	4.94	2.27	38	49.6%	-4.61 [-5.37, -3.85]
Villumsen	2.34	3.22	21	5.22	3.25	20	37.1%	-2.88 [-4.86, -0.90]
**Total (95% CI)**			**89**			**88**	**100.0%**	**-4.68 [-6.90, -2.46]**

Heterogeneity: $Tau^2 = 2.45$; $Chi^2 = 6.77$, df = 2 (P = 0.03); $I^2 = 70\%$
Test for overall effect: Z = 4.13 (P < 0.0001)

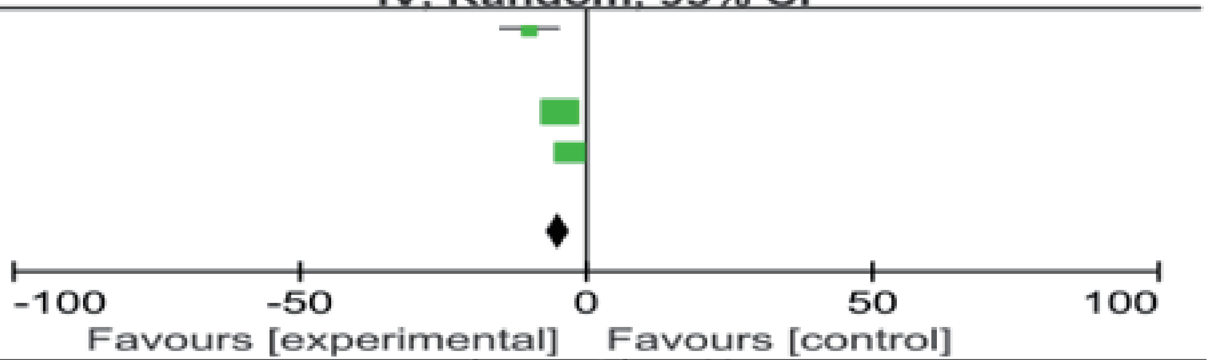

Study or Subgroup	Experimental Mean	Experimental SD	Experimental Total	Control Mean	Control SD	Control Total	Weight	Mean Difference IV, Random, 95% CI
Basha	41.23	10.3	30	51.17	10.45	30	0.0%	-9.94 [-15.19, -4.69]
Feyzioglu	1.53	1.3	19	2.16	1.72	17	33.1%	-0.63 [-1.64, 0.38]
Mohammad	0.33	0.72	38	4.94	2.27	38	58.4%	-4.61 [-5.37, -3.85]
Villumsen	2.34	3.22	21	5.22	3.25	20	8.5%	-2.88 [-4.86, -0.90]
**Total (95% CI)**			**78**			**75**	**100.0%**	**-3.14 [-3.72, -2.57]**

Heterogeneity: $Chi^2 = 38.50$, df = 2 (P < 0.00001); $I^2 = 95\%$
Test for overall effect: Z = 10.65 (P < 0.00001)

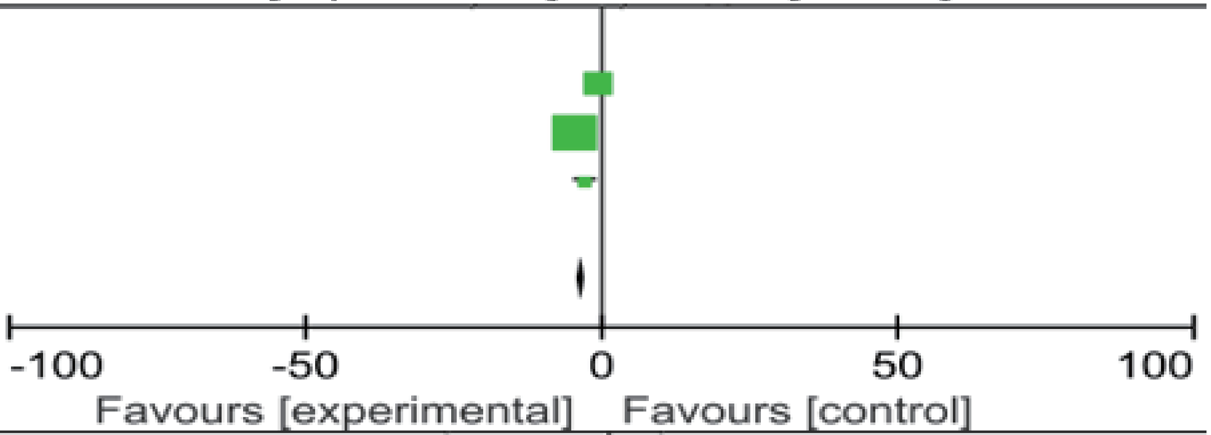

Study or Subgroup	Experimental Mean	Experimental SD	Experimental Total	Control Mean	Control SD	Control Total	Weight	Mean Difference IV, Random, 95% CI
Basha	41.23	10.3	30	51.17	10.45	30	21.2%	-9.94 [-15.19, -4.69]
Feyzioglu	1.53	1.3	19	2.16	1.72	17	41.3%	-0.63 [-1.64, 0.38]
Mohammad	0.33	0.72	38	4.94	2.27	38	0.0%	-4.61 [-5.37, -3.85]
Villumsen	2.34	3.22	21	5.22	3.25	20	37.4%	-2.88 [-4.86, -0.90]
**Total (95% CI)**			**70**			**67**	**100.0%**	**-3.45 [-6.85, -0.04]**

Heterogeneity: $Tau^2 = 7.03$; $Chi^2 = 14.54$, df = 2 (P = 0.0007); $I^2 = 86\%$
Test for overall effect: Z = 1.99 (P = 0.05)

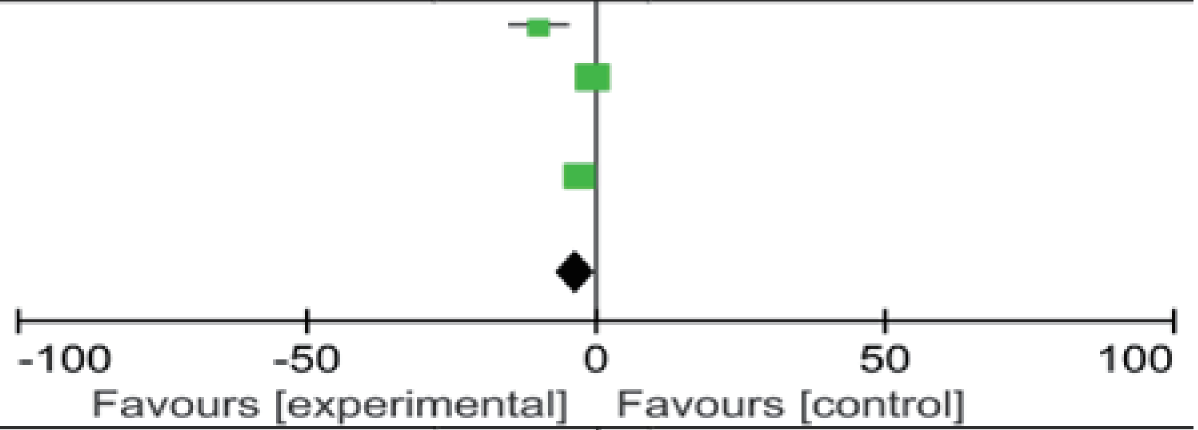

Study or Subgroup	Experimental Mean	Experimental SD	Experimental Total	Control Mean	Control SD	Control Total	Weight	Mean Difference IV, Random, 95% CI
Basha	41.23	10.3	30	51.17	10.45	30	21.7%	-9.94 [-15.19, -4.69]
Feyzioglu	1.53	1.3	19	2.16	1.72	17	38.9%	-0.63 [-1.64, 0.38]
Mohammad	0.33	0.72	38	4.94	2.27	38	39.4%	-4.61 [-5.37, -3.85]
Villumsen	2.34	3.22	21	5.22	3.25	20	0.0%	-2.88 [-4.86, -0.90]
**Total (95% CI)**			**87**			**85**	**100.0%**	**-4.22 [-7.82, -0.61]**

Heterogeneity: $Tau^2 = 8.44$; $Chi^2 = 44.73$, df = 2 (P < 0.00001); $I^2 = 96\%$
Test for overall effect: Z = 2.29 (P = 0.02)

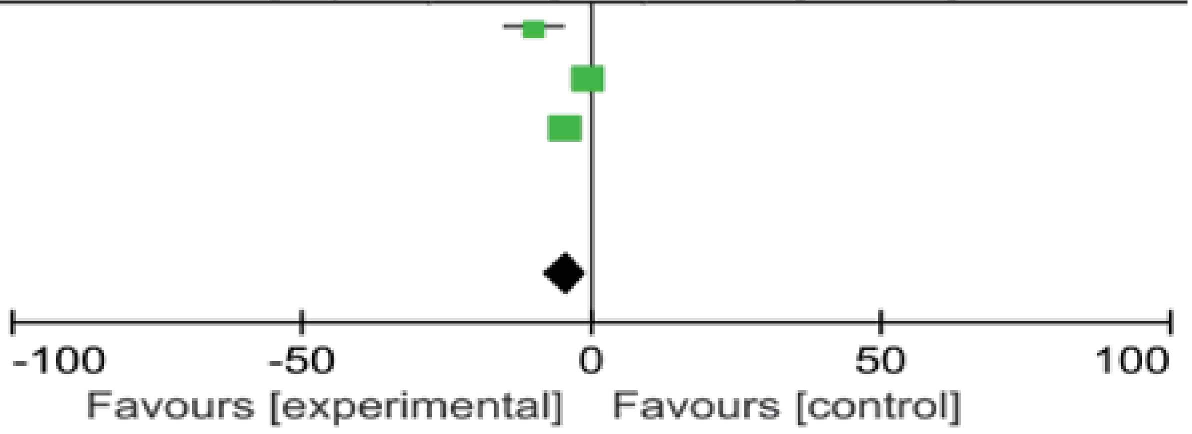

Supplement: Supplemental Information 5 [file peerj-12-18701-s005.pdf]
