# Supplementary figures and images for: Virtual reality therapy in managing cancer pain in middle-aged and elderly: a systematic review and meta-analysis
Source: PeerJ. 2024 Dec 13;12:e18701. doi: 10.7717/peerj.18701 (PMC11648695; doi:10.7717/peerj.18701)

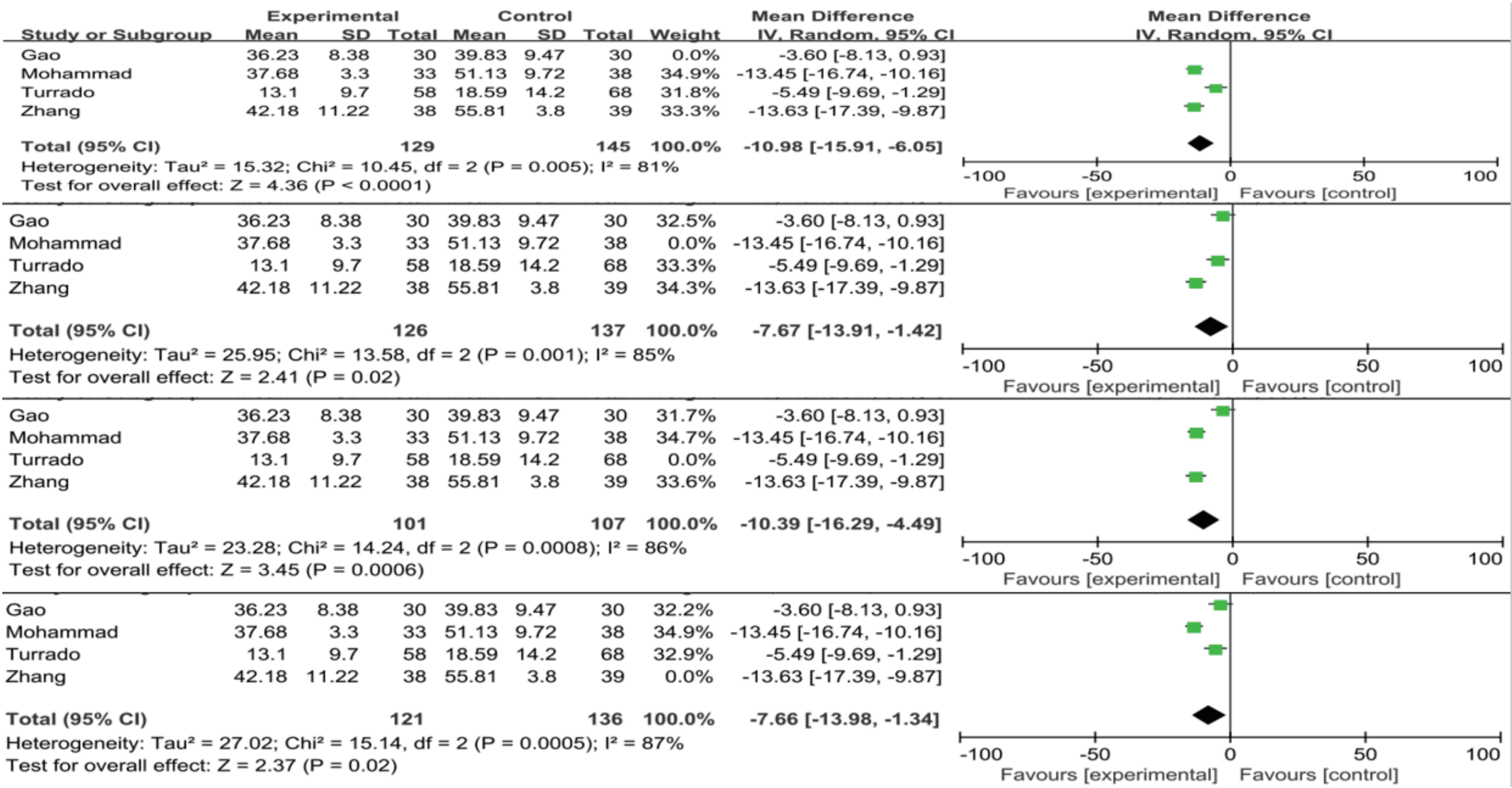

Supplement: Supplemental Information 6 [file peerj-12-18701-s006.pdf]

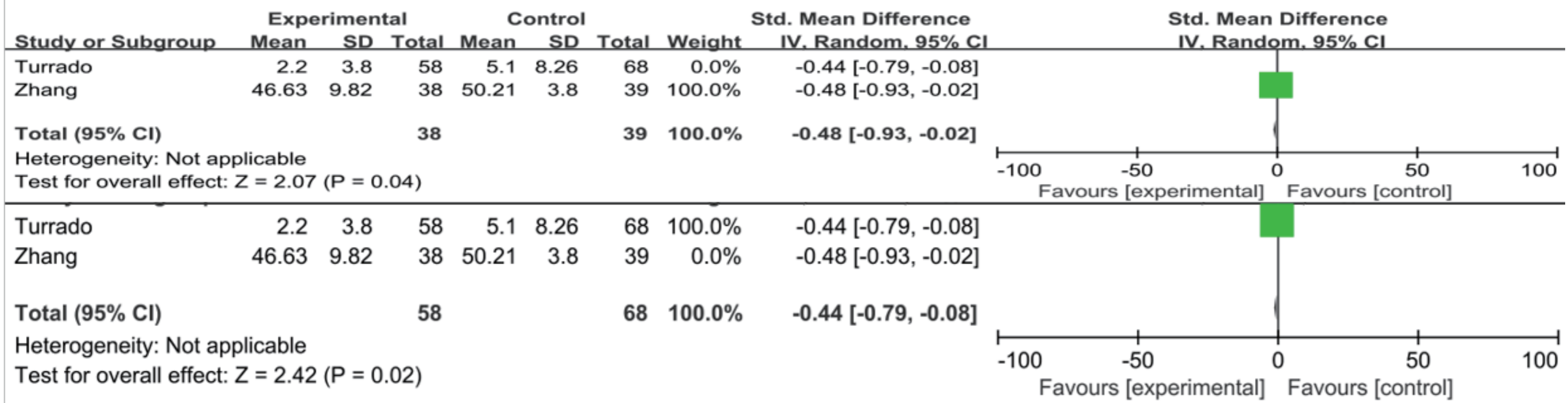

Supplement: Supplemental Information 7 [file peerj-12-18701-s007.pdf]
