# Supplementary material for: Virtual reality therapy in managing cancer pain in middle-aged and elderly: a systematic review and meta-analysis
Source: PeerJ. 2024 Dec 13;12:e18701. doi: 10.7717/peerj.18701 (PMC11648695; doi:10.7717/peerj.18701)
Supplement: Supplemental Information 8 [file peerj-12-18701-s008.docx]

**Search strategy**

PubMed/Scopus/Web of Science

#1.Virtual reality, Cancer [Mesh]

#2. （Virtual reality [Title/Abstract]） OR （VR [Title/Abstract]） OR （Virtual reality scene [Title/Abstract]）OR （Virtual reality game[Title/Abstract]）

#3. （Cancer [Title/Abstract]） OR （tumor[Title/Abstract]）

#4. （Psychological [Title/Abstract]） OR （Symptom [Title/Abstract]） OR （Pain[Title/Abstract]） OR （Anxiety[Title/Abstract]） OR （Depression[Title/Abstract]）

#5.#1 OR #2

#6.#1 OR #2 OR #3

#7.#1 OR #2OR #3OR #4

Cochrane Library

#1. MeSH descriptor: [Virtual Reality, Cancer] explode all trees

#2. (virtual reality): ti, ab,kw OR (/Virtual reality therapy): ti, ab,kw OR (virtual reality scene): ti, ab,kw OR (Virtual reality game): ti, ab,kw

#3. (Cancer):ti,ab,kw OR (tumor):ti,ab,kw

#4. (Psychological):ti,ab,kw OR (Symptom):ti,ab,kw OR (Pain):ti,ab,kw OR (Anxiety):ti,ab,kw OR (Depression):ti,ab,kw

#5.#1 OR #2

#6.#1 OR #2 #3

#7.#1 OR #2OR #3OR #4

Embase database

#1. ‘Virtual reality’/exp, ‘Cancer’/exp

#2. ‘virtual reality’:ti,ab,kw OR ‘Virtual reality therapy’:ti,ab,kw OR ‘virtual reality scene’:ti,ab,kw OR ‘Virtual reality game’:ti,ab,kw

#3. ‘Cancer’:ti,ab,kw OR ‘tumor’:ti,ab,kw

#4. ‘Psychological’:ti,ab,kw OR ‘Symptom’:ti,ab,kw OR ‘Pain’:ti,ab,kw OR ‘Anxiety’:ti,ab,kw OR ‘Depression’:ti,ab,kw

#5.#1 OR #2

#6.#1 OR #2 #3

#7.#1 OR #2OR #3OR #4
