# Supplementary material for: Virtual reality therapy in managing cancer pain in middle-aged and elderly: a systematic review and meta-analysis
Source: PeerJ. 2024 Dec 13;12:e18701. doi: 10.7717/peerj.18701 (PMC11648695; doi:10.7717/peerj.18701)
Supplement: Supplemental Information 9 [file peerj-12-18701-s009.docx]

1. The rationale for conducting the systematic review / meta-analysis

**Respond：** VR technology allows users of computer simulation systems to explore virtual worlds. By using computers to create a simulated environment where users can interact, virtual reality creates an immersive experience that makes it easier for users to feel physically present in the virtual world. In the last several years, virtual reality has gained more traction and acceptance due to the introduction of more affordable gadgets like head-mounted displays. Contrary to most pain relievers, which interfere with the C-fiber route responsible for transmitting harmful signals to the central nervous system (CNS), VR alters the impression of pain by modifying attention, concentration, and emotions.VR creates immersive environments that reduce pain perception by upregulating non-painful brain impulses. As an additional therapeutic alternative for burn pain, acute pain, and experimental pain management in adults, virtual reality is gaining more and more acceptance . Further research has shown that VR lessens pain during various medical procedures, including chemotherapy and wound care, and it has also been proven beneficial when used in conjunction with medications. Individuals experience VR's virtual world, giving them a realistic feeling of being there. The level of presence is determined by the VR aspects displayed to the user. Individuals may develop a psychologically solid belief that they are in the virtual realm rather than the physical world, where pain is experienced. This can occur when they can visually perceive parts of their body in the virtual environment and effectively ignore external distractions from the actual world. Existing literature suggests that VR interventions may be a beneficial method of diversion for chemotherapy patients for various types of cancer. However, there is a scarcity of meta-analyses examining the effectiveness of VR interventions in alleviating cancer pain in elderly patients during chemotherapy. This meta-analysis and systematic review aimed to offer practical guidance on implementing novel alternative approaches for managing cancer pain and assess virtual reality's effectiveness in alleviating cancer pain in older individuals.

1. The contribution that it makes to knowledge in light of previously published related reports, including other meta-analyses and systematic reviews.

**Respond：**This meta-analysis evaluated the effects of VR therapies on symptom management in older adults with cancer. This research suggests that VR substantially impacts the emotional and physical health of older cancer patients. It effectively reduces symptoms of despair, anxiety, and pain. Nevertheless, there was no discernible disparity in the effect on the patient's quality of life. To evaluate the impact of VR on mood regulation and general well-being in older cancer patients, following clinical research should incorporate more significant cohorts of participants and prolong the duration of follow-up in randomized controlled trials. Addressing symptoms in cancer patients is a prolonged process.

.
